# Economic burden of Cardiac Arrest in Spain: analyzing healthcare costs drivers and treatment strategies cost-effectiveness

**DOI:** 10.1186/s12913-023-10274-4

**Published:** 2023-11-07

**Authors:** Mariano Matilla-García, Paloma Ubeda Molla, Fernando Sánchez Martínez, Albert Ariza-Solé, Rocío Gómez-López, Esteban López de Sá, Ricard Ferrer

**Affiliations:** 1 Deparment of Applied Economics and Statistics, UNED, Paseo Senda del Rey, 11, Madrid, 28040 Spain; 2 Cardiology Department. Bellvitge University Hospital. Bioheart. Grup de Malalties Cardiovasculars. Institut d’Investigació Biomèdica de Bellvitge. IDIBELL. L’Hospitalet de Llobregat, Barcelona, 08907 Spain; 3 Intensive Care Unit, Hospital Álvaro Cunqueiro, Vigo, 36312 Spain; 4 Cardiology Service Hospital Universitario La Paz, Pso. de la castellana 261, Madrid, 28046 Spain; 5 Intensive Care department, Hospital Universitari Vall d’Hebron, Barcelona, Spain; 6 Shock, Organ Dysfunction, and Resuscitation (SODIR) Research Group, Vall d’Hebron Institut de Recerca (VHIR) Passeig de la Vall d’Hebron, Barcelona, 08035 Spain

## Abstract

**Background:**

Cardiac arrest is a major public health issue in Europe. Cardiac arrest seems to be associated with a large socioeconomic burden in terms of resource utilization and health care costs. The aim of this study is the analysis of the economic burden of cardiac arrest in Spain and a cost-effectiveness analysis of the key intervention identified, especially in relation to neurological outcome at discharge.

**Methods:**

The data comes from the information provided by 115 intensive care and cardiology units from Spain, including information on the care of patients with out-of-hospital cardiac arrest who had a return of spontaneous circulation. The information reported by theses 115 units was collected by a nationwide survey conducted between March and September 2020. Along with number of patients (2631), we also collect information about the structure of the units, temperature management, and prognostication assessments. In this study we analyze the potential association of several factors with neurological outcome at discharge, and the cost associated with the different factors. The cost-effectiveness of using servo-control for temperature management is analyzed by means of a decision model, based on the results of the survey and data collected in the literature, for a one-year and a lifetime time horizon.

**Results:**

A total of 109 cardiology units provided results on neurological outcome at discharge as evaluated with the cerebral performance category (CPC). The most relevant factor associated with neurological outcome at discharge was ‘servo-control use’, showing a 12.8% decrease in patients with unfavorable neurological outcomes (i.e., CPC3-4 vs. CPC1-2). The total cost per patient (2020 Euros) was €73,502. Only “servo-control use” was associated with an increased mean total cost per hospital. Patients treated with servo-control for temperature management gained in the short term (1 year) an average of 0.039 QALYs over those who were treated with other methods at an increased cost of €70.8, leading to an incremental cost-effectiveness ratio of 1,808 euros. For a lifetime time horizon, the use of servo-control is both more effective and less costly than the alternative.

**Conclusions:**

Our results suggest the implementation of servo-control techniques in all the units that are involved in managing the cardiac arrest patient from admission until discharge from hospital to minimize the neurological damage to patients and to reduce costs to the health and social security system.

**Supplementary Information:**

The online version contains supplementary material available at 10.1186/s12913-023-10274-4.

## Background

Cardiac arrest is a major public health issue in Europe, with an annual incidence of 67 to 170 per 100,000 inhabitants for out-of-hospital cardiac arrest and of 1.5 to 2.8 per 1,000 hospital admissions for in-hospital cardiac arrest; survival rates vary from 0 to 18% for out-of-hospital cardiac arrest and 15–34% for in-hospital cardiac arrest [1]. Although research on this area is somewhat limited, cardiac arrest seems to be associated with a large socioeconomic burden in terms of resource utilization and health care costs, 2]. with inpatient management as the main driver of health care cost regardless of the age of the patient [2, 3].

Postresuscitation care plays a key role in survival and improving neurological outcomes. According to the 2021 guidelines from the European Resuscitation Council and European Society of Intensive Care Medicine, postresuscitation care includes control of oxygenation and ventilation, hemodynamic optimization, coronary reperfusion, targeted temperature management, control of seizures, prognostication, and rehabilitation [4]. Among these measures, temperature control has been cardinal since it has shown a benefit in terms of neurological outcomes [5, 6]. Real-world data suggest that resuscitation guidelines could contribute to improving survival at discharge and neurological outcomes [7]. However, adherence to guideline recommendations and postresuscitation practices vary greatly across countries [8] and even across centers within the same country [9].

In this context, the CAPAC project (*Certificación Asistencial en Paro Cardíaco* - Accreditation in Cardiac Arrest Care) was created with the aim of improving hospital care for patients with cardiac arrest through the accreditation of cardiac resuscitation units in Spanish hospitals. This initiative was endorsed by the Spanish Society of Intensive Care (SEMYCIUC) and the Spanish Cardiology Society (SEC). As part of this project, we conducted a nationwide survey on postcardiac arrest management across the different intensive care and cardiology units of hospitals to ascertain the variations in clinical practice in Spain. The results of this survey can be found elsewhere [10]. Using data from this survey, we present herein an analysis of the economic burden of cardiac arrest and a cost-effectiveness analysis of the key intervention identified, especially in relation to neurological outcome at discharge.

## Methods

### The CAPAC survey

The CAPAC survey was conducted between March and September 2020 among 115 Spanish centers. It comprised general information such as hospital, autonomous community, city, type of unit, and number of patients with out-of-hospital cardiac arrest admitted to each hospital per year. It had 34 questions divided into 4 sections: care of patients with out-of-hospital cardiac arrest who had a return of spontaneous circulation, the structure of the unit, temperature management, and prognostication. Among the prognostication items, neurological function at discharge was described by Performance Category (CPC) score. CPC categories are the following [11]: CPC1 (good cerebral performance), conscious, alert, able to work and lead a normal life; CPC2 (moderate cerebral disability), conscious, sufficient cerebral function for part-time work in sheltered environment or independent activities of daily life (dress, travel by public transportation, food preparation); CPC3 (severe cerebral disability), conscious but dependent on others for daily support (in an institution or at home with exceptional family effort) and at least limited cognition; CPC4 (coma/vegetative state), unconscious, unaware of surroundings, no cognition, no verbal or psychological interaction with the environment; and CPC5 (brain death), certified brain dead or dead by traditional criteria. Given de sanitary circumstances, we decided to stop the data collection ahead of the schedule, reaching 83% of the total national representativeness. However, the responses to the survey questions were not affected by the effect of the pandemic, as they refer to the structures and protocols followed by hospitals in times of normality. The complete survey is available in Supplementary Table 1.

### Multivariate analyses

The potential association of several factors with neurological outcome at discharge was analyzed by means of a fractional multinomial logit model. The CPC score was dichotomized into ‘favorable’ (CPC 1 and 2) and ‘unfavorable’ (CPC 3 and 4) outcomes and used as the dependent variable. This model shows the associated effects of the considered variables on the percentage of patients in the different neurological states. Particularly, this model aims to show the associated effects on the proportion of patients with unfavorable states compared to those with favorable states. We also ran another fractional multinomial logit model using as the base outcome the neurological outcome of CPC2 and we compared with CPC3; it seems reasonable to consider a greater likelihood of changing from state CPC3 to CPC2 than from CPC4 to CPC2 or CPC1. We aimed to capture the differential effects of the explanatory variables on the proportion of patients in states CPC 2 and CPC 3. The independent variables for both models were selected based on clinical grounds and included coronarography and percutaneous coronary intervention (PCI), time objective for PCI, PCI execution time, targeted-temperature management (TTM) active control, time objective for TTM initiation, use of servo-control temperature systems, thermal recovery, “the aim is to reach a temperature”, use of prognostic scales during the first 72 h, long-term monitoring, and rehabilitation program. The definitions of each of these variables are shown in Supplementary Table 2.

For the analysis of factors associated with the mean total cost of cardiac arrest per hospital (see calculation below), we used a multiple linear regression model with the total cost in euros as the dependent variable and the same explanatory variables mentioned above for the neurological outcome model.

### Cost estimation per patient

To calculate the cost per patient of their stay in the ICU, we used the data of the average stay of patients with cardiac arrest in the ICU of the 109 hospitals and multiplied this by the average price of each day (average stay) they spent in this unit. We used the same procedure for the hospitalization cost per patient. To calculate the cost per patient of carrying out a prognostication, we used the average cost of the different methods used in these 109 hospitals to carry out the prognostication of their patients (neurological examination, neuron-specific enolase, magnetic resonance imaging, computer tomography, electroencephalogram, somatosensory evoked potentials). The average cost per patient of the rehabilitation phase was estimated as the average of the cost of rehabilitation for each of the neurological outcomes. The cost per session and the number of rehabilitation sessions increase according to the neurological outcome, being lower in CPC1 and higher in CPC4. Therefore, these data were obtained by multiplying the price per session by the average number of sessions for each neurological outcome and then calculating the average of these four costs. The cost of temperature control included both the cost of inducing hypothermia and the rewarming cost. The cost of the hypothermia phase was the average cost of this phase. That is, we estimated the average cost of using the different temperature control procedures (servo-control measures, crystalloids, antipyretic medication, etc.). It should be noted that rewarming is only carried out on some patients, whereas others are rewarmed passively, which does not incur any costs. Finally, the average indirect costs per patient are the average of the indirect costs of patients with CPC2, CPC3 and CPC4, which are higher as the neurological outcome becomes poorer. Indirect costs estimate the losses related to labor productivity (as losses of productivity of the patient and of the household and due to losses of leisure activities) [12–14].

### Cost-effectiveness analysis of servo-control for temperature management

As only controlling the temperature through the use of servo-control had a significantly positive effect on the proportion of patients with improved neurological outcome at discharge (see below), we analyzed the cost-effectiveness of the use of this technique.

**Fig. 1:**
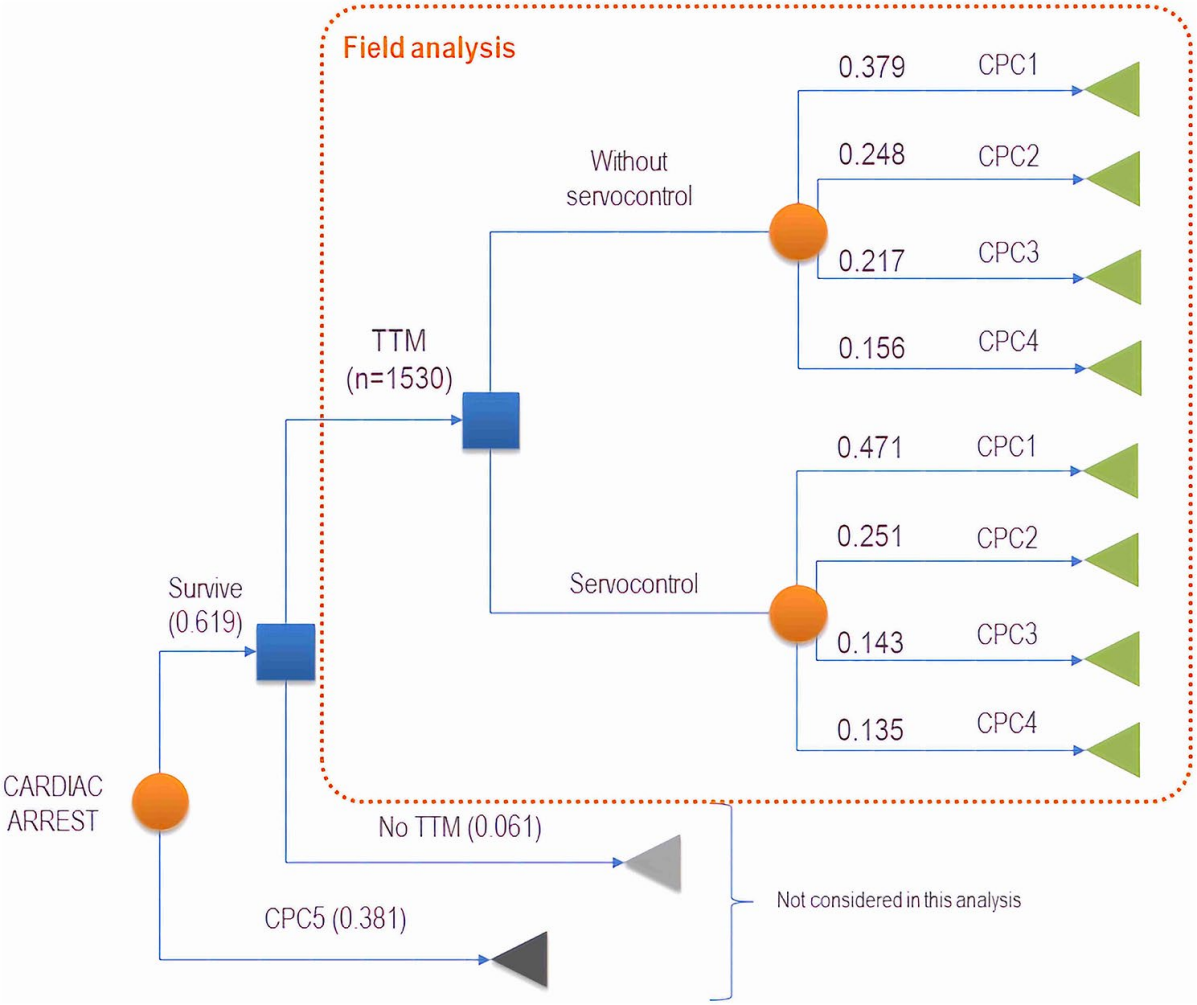
Decision model structure

We created a decision model based on the results of the CAPAC survey to follow a cohort of 1,530 patients who survived an out-of-hospital cardiac arrest and were admitted to the hospital (Fig. 1).

### CPC, Cerebral Performance Category; TTM, targeted-temperature management

Cost calculations were made as mentioned above. The expected values of the indirect costs (lifetime) were obtained by applying the probabilities corresponding to each treatment strategy in the decision tree (Fig. 1) to the indirect costs according to the CPC as calculated in our analysis and then adding those expected annual values for the horizon determined by the life expectancy, according to neurological outcome as reported in the literature [15] (see also Table 1), with a discount rate of 3%.

**Table 1:** Base case variables for the cost-effectiveness model of using servo-control systems for controlling temperature

Variable	Base case	Source
Probability of survival among patients with cardiac arrest admitted to hospital	0.619	CAPAC survey
Probability of neurological outcome according to the use of servo-control for temperature management		CAPAC survey and data normalized according to the number of patients treated with the method of temperature control
With servo-control		
CPC1	0.379	
CPC2	0.248	
CPC3	0.217	
CPC4	0.156	
Without servo-control		
CPC1	0.471	
CPC2	0.251	
CPC3	0.143	
CPC4	0.135	
Direct costs (€/patient and year)		CAPAC survey
With servo-control		
CPC1	27.964	
CPC2	30.505	
CPC3	33.581	
CPC4	36.952	
Without servo-control		
CPC1	28.634	
CPC2	31.175	
CPC3	34.251	
CPC4	37.622	
Indirect costs (€/patient and year)		CAPAC survey
CPC1	0	
CPC2	22.077	
CPC3	37.259	
CPC4	60.633	
Utility (quality weight)		Gajarski et al [16]
CPC1-CPC2	0.76	
CPC3-CPC4	0.35	
Life expectancy (years)		Coute et al [15]
CPC1-CPC2^a^	12.5	
CPC3-CPC4^b^	8.0	

CPC, Cerebral Performance Categor

^a^Patients discharged to home

^b^Patients discharged to hospice

The effectiveness of controlling temperature with or without servo-control was also based on the literature [16]. In the short term (1 year), the expected values were obtained by applying the probabilities corresponding to each treatment strategy on the decision tree (Fig. 1) to the utilities or quality weights we summarized in Table 1. In the long term, these annual expected values were added for the horizon determined by the life expectancy, according to neurological state (Table 1), with a discount rate of 3%.

Our final model used a societal perspective, including both direct and indirect costs.

## Results

### Overall results of the CAPAC survey

Among the 115 respondents, 109 provided results on neurological outcome at discharge as evaluated with the CPC. Of the 2,631 patients per year seen by the units, it was estimated by the respondents that 62% were alive at discharge, distributed as 44% with good cerebral performance (CPC1), 25% with moderate cerebral disability (CPC2), 17% with severe cerebral disability (CPC3) and 14% with coma or vegetative state (CPC4) (Supplementary Fig. 1). Further information on the answers from centers and their main characteristics are in the Supplementary section (see ST 6 to ST 9).

### Factors associated with neurological outcome at discharge

In the two fractional multinomial models, the only factor associated with neurological outcome at discharge was the use of servo-control (Table 2). Using catheters/hydrogel patches with advanced servo-control devices compared with alternative methods was associated with a 12.8% decrease in patients with unfavorable neurological outcomes (i.e., CPC4 or CPC3 compared to neurological favorable states) at discharge (p < 0.05).

**Table 2:** Marginal effects of the fractional multinomial logit model

Variables	Unfavorable CPC vs. Favorable CPC Coefficient (SE)	p-value	CPC3 vs. CPC2 Coefficient (SE)		p-value
Coronarography and PCI	0.078 (0.064)	0.221	-0.019 (0.039)		0.622
Time objective for PCI	-0.032 (0.067)	0.624	0.006 (0.051)		0.911
PCI execution time	-0.003 (0.000)	0.571	-0.001 (0.000)		0.287
TTM active control	-0.130 (0.119)	0.274	-0.151 (0.120)		0.209
Time objective for TTM initiation	0.039 (0.060)	0.513	-0.021 (0.039)		0.586
Use of servocontrol	-0.128** (0.064)	0.047	-0.098** (0.043)		0.023
Thermal recovery	0.039 (0.080)	0.625	0.011 (0.061)		0.854
The aim is to reach a temperature	-0.058 (0.086)	0.496	-0.039 (0.080)		0.625
Use of prognostic scales 72 h	-0.090 (0.056)	0.105	-0.036 (0.038)		0.325
Long-term monitoring	-0.094 (0.056)	0.095	-0.031 (0.038)		0.380
Rehabilitation program	-0.005 (0.060)	0.926	0.012 (0.041)		0.757
Observations	79			79	

CPC, Cerebral Performance Category; PCI, percutaneous coronary intervention; TTM, targeted-temperature management; **p < 0.05

The results of the marginal effects of the explanatory variables on the proportion of patients in states CPC2 and CPC3, which uses state CPC2 as a basis for comparison, corroborate that the use of the servo-control generates a decrease in the percentage of patients in state CPC3. In particular, the application of this temperature control technique would reduce patients in state CPC3 by 9.8% compared to the proportion of patients in neurological state CPC2.

When normalized based on the number of patients treated with or without servo-control of the temperature, the proportion of patients with a favorable neurological state at discharge was 72.2% with servo-control of the temperature and 62.7% without servo-control of the temperature (Supplementary Table 3).

### The cost associated with Cardiac Arrest

The total cost per patient (2020 Euros) with out-of-hospital cardiac arrest admitted to the hospital was €73,502, with indirect costs contributing to 54.4% of the total cost (Table 3). Among direct costs, ICU and rehabilitation costs were the largest contributors, with almost 15% each of the total costs, and the TTM contributed 3.5% of the total costs. When analyzed by CPC neurological outcome, the cost per patient increased as the outcome became poorer, with a cost of €28,332.7 for patients with a CPC1 score and €97,953.1 for patients with a CPC4 score (Supplementary Table 4).

**Table 3:** Average direct and indirect costs per patient admitted to the hospital with cardiac arrest

Average cost per patient	2020 Euros	% of the total cost
Average direct cost	33,513	45.6
Average ICU cost	11,001 (7,666)	15.0
Average cost for hospital stay	8,906 (6,933)	12.1
Average prognostication cost	210 (59)	0.3
Average rehabilitation cost	10,878 (0)	14.8
Average TTM cost^a^	1,809 (1,588)	3.5
Average indirect cost	39,989	54.4
Average total cost	73,502	100

ICU, intensive care unit; TTM, targeted-temperature management

^a^Average TTM cost refers to the cost of inducing hypothermia and rewarming

The mean total cost per hospital was €768,474, with direct costs comprising 60.5% of the total cost (Supplementary Table 5); ICU, hospital stay, and rehabilitation costs were the major contributors to the total cost and contributed to a similar extent. In the multiple linear regression analysis (Table 4), the single factor significantly associated with an increased mean total cost per hospital was ‘servo-control use’ (β 472,142), while the variable ‘the aim is to reach a temperature’ was significantly associated with a decreased mean total cost per hospital (β -601,655).

**Table 4:** Multiple linear regression analysis of the factors associated with the mean total hospital cost associated with cardiac arrest

Variable	β coefficient	p value
Coronarography and PCI	13,416. (248,573)	0.957
Time objective for PCI	-377,917 (260,291)	0.151
PCI execution time	-195 (1,299)	0.881
PCI availability	349,565 (229,044)	0.131
TTM active control	-74,791 (253,659)	0.769
Time objective for TTM initiation	318,971 (173,295)	0.069
Use of servocontrol	472,142** (214,574)	0.031
Thermal recovery	64,263 (171,226)	0.708
The aim is to reach a temperature	-601,655** (287,616)	0.04
Use of prognostic scales 72 h	97,538 (190,784)	0.611
Long-term monitoring	358,509 (186,331)	0.058
Rehabilitation program	262,133 (165,354)	0.117
Constant	772,754 (387,997)	0.05
R [2] Observations	0.3173 79	

PCI, percutaneous coronary intervention; TTM, targeted-temperature management. Standard errors in parentheses. **p < 0.05

### Cost-effectiveness analysis of using servo-control systems for temperature management

Using our base case (Table 1), patients treated with servo-control for temperature management gained in the short term (1 year) an average of 0.039 Quality Adjusted Life Year (QALYs) over those who were treated with other methods at an increased cost of €70.8, leading to an incremental cost-effectiveness ratio of 1,808.04 euros (Table 5). When using a lifetime horizon, patients treated with servo-control for temperature management gained 0.563 QALYs at a decreased cost of €31,600 (Table 5).

**Table 5:** Results of the cost-effectiveness analysis over the lifetime expectancy horizon

	With servo-control (A)	Without servo-control (B)	Incremental (A)-(B)
Effectiveness (average QALY)			
1 year	0.646	0.607	0.039
Lifetime	7.067	6.505	0.563
Average cost (€)			
1 year (direct cost)	31,285.28	31,214.53	70.76
Indirect cost (lifetime)	171,681.47	203,352.00	-31,670.53
Total lifetime^a^	202,966.76	234,566.53	-31,599.67
Incremental cost-effectiveness ratio (€/QALY gained)			
1 year (only direct costs)	---	---	1,808.04
Total lifetime^a^	---	---	Dominant^b^

^a^Total lifetime average cost included the short-term and indirect cost for life expectancy. ^b^Strategy A (“with servo-control”) is both clinically superior and cost saving

QALY, Quality-Adjusted Life-Year

## Discussion

The analysis of this survey conducted in a large sample of Spanish centers shows that the major factor for having a better neurological outcome at discharge is the use of servocontrol for temperature management. This factor was also significantly associated with an increased total cost per hospital. However, it was a cost-effective measure with a low incremental cost-effectiveness ratio.

The focus of the management of cardiac arrest should be improving survival, as well as neurological outcomes and quality of life among survivors [17]. To this end, the European Resuscitation Council and European Society of Intensive Care Medicine recommend several postresuscitation care measures that include targeted temperature management [4]. In the CAPAC survey, we found that, excluding patients with brain death, the participants reported that 31% of the patients had an unfavorable neurological outcome at discharge (i.e., a CPC3 or CPC4). In the fractional multinomial model, we found that, when compared with alternative methods, the use of servo-control for temperature management was the single factor significantly associated with the neurological outcome, with a 12.8% decrease in patients with unfavorable neurological outcomes. This is consistent with the current evidence and current recommendations of using controlled methods for the induction (in combination with conventional methods or not) and maintenance of temperature control [18]. We are not aware of other studies reporting the specific management of cardiac arrest as a predictor of neurological outcome. Sandroni et al., [19] in a systematic review of the literature, reported that some clinical, biomarker, electrophysiology, and imaging variables were significantly associated with a good neurological outcome; similarly, some clinical, biochemical, neurophysiological, and radiological variables have been associated with a poor neurological outcome [20]. Target temperature management has been the cornerstone of cardiac arrest management due to its association with neurological outcomes. However, recent randomized trials and a systematic review have changed the paradigm, [5, 21] and currently, the European Resuscitation Council and European Society of Intensive Care Medicine have issued new recommendations regarding temperature control in 2022, where they recommend continuing to monitor core temperature and actively prevent fever for at least 72 h, but they did not find enough evidence for or against temperature control at 32–36 °C [6].

We found that the total cost per patient was €73,502, with the total cost increasing as the neurological outcome became poorer; in fact, the cost of patients discharged at CPC4 was over threefold the cost of those discharged at CPC1 (€97,953.1 vs. €28,332.7). These results are consistent with those of a single center conducted in a tertiary hospital in Finland [22]. In that study, the author analyzed the health care-associated cost with 1-year survival for patients with cardiac arrest treated at the intensive care unit and found that total cost was lower for survivors with a favorable neurological outcome than for those with an unfavorable outcome regardless of the locations of the cardiac arrest or the initial cardiac rhythm [22]. Thus, for patients with an out-of-hospital cardiac arrest, the mean cost per patient with a CPC1-2 at one year was €59,813 and for those with a CPC3-4 was €104,147 [22].

In the multivariate model, the use of servo-control for temperature management was associated with a significantly higher cost than alternative methods. The management of temperature in patients with cardiac arrest has also been associated with significantly higher cost in other studies. The analysis of the model also suggests that both having an initial temperature control objective and using servo-control techniques significantly increase the costs borne by hospitals. However, these can be reduced if there is a declared and protocolized target level. These findings are compatible with previous ones. Geri et al., [2] in a study of the total costs associated with the management of out-of-hospital cardiac arrest in a large Canadian health system, reported that among the factors independently associated with cost was targeted temperature management, with a rate ratio of 1.25 (95% CI 1.09 to 1.44). Using the US Nationwide Inpatient Sample, Damluji et al [23] analyzed the costs associated with index hospitalizations after cardiac arrest in the United States and found that, among selected interventions, hypothermia was significantly associated with an increased cost with an odds ratio of 1.28.

Although the use of servo-control for temperature management was associated with higher cost, in our cost-effectiveness model, it was also associated with a gain of 0.039 QALYs compared to alternative methods at an increased cost of €70.8 and a cost-effectiveness ratio of €1.804. This cost-effectiveness ratio is below the lower limit of the UK National Institute for Health and Clinical Excellence (NICE) cost-effectiveness threshold range (i.e., 20,000 pound sterling) and the threshold reported for the National Health System in Spain (i.e., between €22,000 and €25,000) [24]; therefore, this intervention could be considered cost-effective. Moreover, in a lifetime horizon, according to our model, patients treated with servo-control for temperature management gained 0.563 QALYs at a decreased cost of €31,600.

In terms of the implications of our findings for Public Health, it could be advisable for units that are involved in managing the cardiac arrest patient from admission until discharge from hospital to follow the recommendations on post-resuscitation care. Of particular importance would be the implementation of servo-control techniques in all these units to minimize the neurological damage to patients and to reduce costs to the health and social security system.

The major limitation of our study was the use of a survey for estimating clinical outcomes. However, this survey exhibits notable peculiarities that should be considered to limit the scope of the consequences of its use. Please, notice that the survey was based on a large sample of hospitals in Spain and notice also that the respondents were those responsible for the management of cardiac arrest in each involved unit. Respondents possess direct knowledge of their hospitals’ protocols as well as detailed information about their patients’ progress. Therefore, their responses were based on the information of their departments/units. It is also noteworthy that, unlike other types of surveys, the respondents are not the subjects under treatment, which significantly reduces potential biases attributed to this aspect.

It is also remarkable that the survey was not an opinion survey; it is a highly detailed technical survey in which the questions and potential responses (accessible in the appendix) were designed to minimize potential biases often associated with general surveys. Nevertheless, working with aggregated data, rather than individual registration data (patient data), results in a loss of heterogeneity. This loss of individual variation makes the study of confounding factors difficult.

Another problem associated with non-randomized studies is selection bias of patient to treatments. Although this problem is present in this study, its severity is reduced by the derivation process followed by the ambulance emergency services. In this study, subjects (patients) are assigned to different hospitals, and consequently, to various processes in the treatment of cardiac arrest, based on the distance between the location where the cardiac arrest occurs and the hospital. This quasi-random assignment (by design) significantly reduces the risk of the exposed models suffering from potential confounding unobserved variables and also from potential selection bias. The approach that could potentially attempt to mitigate the impact of those potential risks would be to use Randomized Controlled Trials (RCTs), despite the ethical difficulties that would entail. Therefore, the results of this study cannot necessarily be interpreted as causal. Conducting a similar study based on administrative data would be advisable.

It is also interesting to note that in our cost analysis we have not considered patients in the CPC5 state. In the case of temperature management, it is understood that the patient who ends up in CPC5 does so regardless of the temperature control mechanism used.

Overall, our results suggest that the use of servo-control for temperature management is associated with better neurological outcomes and, although it is associated with a higher cost, is a cost-effective measure.

## Conclusions

Our results suggest that, once taken into account the effects of several well-stablished factors associated with neurological outcome at discharge, the use of servo-control for temperature management is associated with better neurological outcomes and, although it is associated with a higher cost, it can be evaluated as a cost-effective measure.

### Electronic supplementary material

Below is the link to the electronic supplementary material.

Supplementary Material 1

## Data Availability

The datasets generated and/or analysed during the current study are not publicly available because are property of two medical societies, SEMYUC and SEC. However, they are available from the corresponding author on reasonable request.
