# Supplementary material for: Economic burden of Cardiac Arrest in Spain: analyzing healthcare costs drivers and treatment strategies cost-effectiveness
Source: BMC Health Serv Res. 2023 Nov 7;23:1220. doi: 10.1186/s12913-023-10274-4 (PMC10631046; doi:10.1186/s12913-023-10274-4)
Supplement: Supplementary file 1 — Supplementary Material 1 [file 12913_2023_10274_MOESM1_ESM.docx]

**Supplementary material for: Economic burden of cardiac arrest in Spain: Analyzing healthcare costs drivers and treatment strategies cost-effectivenes**

**Supplementary Table 1**

**CAPAC Questionnaire**

| **GENERAL INFORMATION** |
| --- |
| Name of the hospital: ___________________(deductive drop-down) the drop-down menu needs to be added |
| Autonomous Community ________________(deductive drop-down) |
| City: ___________________ (predictive drop-down) |
| What is your unit? _______________ |
| o Cardiac |
| o Intensive/multi-purpose |
| Number of beds in your Unit: ________ |
| Number of admissions of comatose patients for out-of-hospital cardiac arrest per year: _________ |
|  |
| **CARE OF COMATOSE PATIENTS AFTER OUT-OF-HOSPITAL CARDIAC ARREST (CRA) WITH RETURN OF SPONTANEOUS CIRCULATION (ROSC)** |
|  |
| **1. Do you have a post-cardiac arrest protocol?** |
| a. Yes |
| b. No |
| **2. Please indicate which protocols you have in place in your Unit: (Multiple answers)** |
| a. Referral protocols |
| b. Diagnostic approach protocol |
| c. Temperature control |
| d. Neurological prognosis |
| e. Organ donation |
| **Rank the selected options from most to least important: __________** |
| **3. Which hospital department receives comatose patients recovered from suspected non-cardiac out-of-hospital cardiac arrest (OHCA)?** |
| a. Emergency service |
| b. Cardiac catheterisation laboratory |
| c. ICU/Critical Unit |
| d. Other: _______________ |
| **4. Which hospital service receives comatose patients recovered from suspected cardiac OHCA?** |
| a. Emergency service |
| b. Cardiac catheterisation laboratory |
| c. ICU/Critical Unit |
| d. Other: ______________ |
| **5. In which patient populations do you perform coronary angiography and emergent percutaneous coronary intervention (PCI)?** |
| a. None |
| b. All |
| c. Some |
| **Which ones? (Multiple answers)** |
| a. Elevated ST only |
| b. Any suspicion of acute coronary syndrome |
| c. Unexplained CRA |
| d. Refractory cardiac arrest |
| e. Clinical instability |
| **6. Is there an age limit for not performing coronary angiography and emergent PCI in comatose patients?** |
| a. Yes |
| b. No |
| **If yes, please indicate what are: __________** |
| **7. In ST-elevation acute coronary syndrome (STEACS), do you have a time target for PCI?** |
| a. Yes |
| b. No |
| **If yes, is it routinely measured?** |
| a. Yes |
| b. No |
| **If so, what is the time? ____h_____min** |
| **8. In actual clinical practice, what is the average time for PCI?** |
| a. 60 minutes |
| b. 120 minutes |
| c. 180 minutes |
| d. 240 minutes |
| e. 300 minutes |
| f. 360 minutes |
| **9. Do you carry out regular quality audits?** |
| a. Yes |
| b. No |
| **10. Do you have a screening protocol for arrhythmogenic diseases?** |
| a. Yes, electrophysiological/pharmacological study |
| b. Yes, genetic study |
| c. Both |
| d. No |
| **STRUCTURE** |
| **11. Do you have a PCI service in your centre?** |
| a. Yes, 24 Hours, 7 days a week |
| b. Yes, but with restricted shift |
| c. I do not have this service |
| **If you select B, please answer the following question:** |
| **Please indicate which:__________________** |
| **12. Do you have an ultrasound service in your centre?** |
| a. Yes, 24 Hours, 7 days a week |
| b. Yes, but with restricted shift |
| c. I do not have this service |
| **If you select B, please answer the following question:** |
| **Please indicate which:__________________** |
| **13. Do you have a CT service in your centre?** |
| a. Yes, 24 Hours, 7 days a week |
| b. Yes, but with restricted shift |
| c. I do not have this service |
| **If you select B, please answer the following question:** |
| **Please indicate which:__________________** |
| **14. Do you have an EEG service in your centre?** |
| a. Yes, 24 Hours, 7 days a week |
| b. Yes, but with restricted shift |
| c. I do not have this service |
| **If you select B, please answer the following question:** |
| **Please indicate which:__________________** |
| **15. Do you have a neurophysiology service in your centre?** |
| a. Yes, 24 Hours, 7 days a week |
| b. Yes, but with restricted shift |
| c. I do not have this service |
| **If you select B, please answer the following question:** |
| **Please indicate which:__________________** |
| **16. Do you have a cardiology service in your centre?** |
| a. Yes, 24 Hours, 7 days a week |
| b. Yes, but with restricted shift |
| c. I do not have this service |
| **If you select B, please answer the following question:** |
| **Please indicate which:__________________** |
| **TEMPERATURE CONTROL MANAGEMENT** |
| **17. Do you actively monitor the temperature of comatose patients after out-of-hospital cardiac arrest with ROSC?** |
| a. Yes, for all patients |
| b. Yes, only in patients with shockable rhythm. |
| c. No |
| **18. Where does temperature control (TTM) start?** |
| a. Emergency service |
| b. Cardiac catheterisation laboratory |
| c. ICU |
| d. Other: _______________ |
| **19. Is TTM initiated before or after PCI?** |
| a. Before PCI |
| b. During PCI |
| a. After PCI |
| **20. Do you have a time target for the start of TTM?** |
| a. Yes |
| b. No |
| **If so, what is the time? ____h_____min** |
| **If yes, is it routinely measured?** |
| a. Yes |
| b. No |
| **21. How do you monitor the temperature? (Multiple answers)** |
| a. It is not generally monitored |
| b. Antipyretic medication |
| c. Cold fluids/crystalloids |
| d. Physical measures: cold compresses, fans/wet towels |
| e. Non-feedback sheet/water mattress (without servo-control) |
| f. Hydrogel catheters/patches with advanced servo-control devices |
| **22. Do you perform temperature recovery after TTM?** |
| a. Yes |
| b. No |
| **If so, what is the time? (Drop-down)** |
| a. <0.1oC/hour |
| b. 0.1 – 0.25oC/hour |
| c. 0.26 – 0.5oC/hour |
| **23. Do you have a written TTM protocol?** |
| a. Yes |
| b. No |
| **24. Does it have a target temperature?** |
| a. Yes |
| b. No |
| **If yes:** |
| **a. It is a fixed temperature: _______** |
| **b. It operates in a range: minimum____ maximum____** |
| **PROGNOSIS** |
| **25. Do you apply prognostic scales in the first 72 hours?** |
| a. Yes |
| b. No |
| **If yes, please indicate which: __________** |
| **26. When is neuroprognosis performed? (Multiple answers)** |
| a. 72 hours after the arrest |
| b. Immediately after rewarming |
| c. 72 hours after rewarming |
| d. I don't have a set time |
| **27. Do you have a protocol to limit therapeutic effort?** |
| a. Yes |
| b. No |
| **If yes, do you consider donation in end-of-life care?** |
| a. Yes |
| b. No |
| 28. **Indicate which prognostic method you use (Multiple answers)** |
| a. Neurological examination |
| b. Neuro-specific enolase |
| c. MRI |
| d. CT |
| e. EEG |
| f. Somatosensory evoked potentials |
| g. Others: _____________ |
| **29. Indicate which assessments are made in the neurological assessment upon discharge (Multiple answers)** |
| a. mRS (Modified Rankin Scale) |
| b. CPC (*cerebral performance category*) |
| c. GOS (Glasgow Outcome Scale) |
| d. Pupillary reflex |
| e. Corneal reflex |
| **30. Is there long-term follow-up of patients?** |
| a. Yes |
| b. No |
| **If yes, where is it done? (Hospital/Health Centre) _______** |
| **If yes, how often? (months) _______** |
| **31. Do you have a rehabilitation protocol for patients?** |
| a. Yes |
| b. No |
| **If yes, please indicate which: (Multiple answers)** |
| a. Locomotive rehabilitation |
| **What is the average number of sessions you perform? ______** |
| **What is the average duration of rehabilitation? (months)_____** |
| b. Neuro-rehabilitation |
| **What is the average number of sessions you perform?______** |
| **What is the average duration of rehabilitation? (months)_____** |
| c. Occupational therapy |
| **What is the average number of sessions you perform?______** |
| **What is the average duration of rehabilitation? (months)_____** |
| **32. Indicate the percentage of patients per year according to neurological damage on discharge:** |
| a. CPC1 (without sequelae)___________ |
| b. CPC2 (mild disability, is independent, does not require institutionalisation)__________ |
| c. CPC3 (severe disability, not independent, requires institutionalisation) ________ |
| d. CPC4 (persistent vegetative state)___________ |
| e. CPC5 (death)________ |
| **33. Indicate, in days, the average length of stay:** |
| a. In the ICU/CU _________ |
| b. In the hospital ________ |
| **34. Would you be interested in participating in a prospective register of cardiac arrest management?** |
| a. Yes |
| b. No |

**Supplementary Table 2. Explanatory variables used in the multivariate models**

| **Explanatory variable** | **Definition** |
| --- | --- |
| Coronarography and PCI | A value of 1 is given if all patients undergo coronary angiography and PCI and a 0 in all other cases. |
| Time objective for PCI | A value of 1 is given if the procedure anticipates a time objective for performing the PCI and it is normally measured, and a 0 if otherwise. |
| PCI execution time | Average time in which PCI is performed. Precoded into one hour periods in the survey. A value of 1 is given if the average time during which PCI is performed is 60 minutes, 2 if it is 120 minutes, 3 if it is 180 minutes, 4 if it is 240 minutes, 5 if it is 300 minutes and 6 if it is 360 minutes. |
| PCI availability | A value of 1 is given if there is a 24 hour PCI service, and a 0 for all other options of the reference question. |
| TTM active control | A value of 1 is given if the temperature is controlled actively and a 0 if it is not. |
| Time objective for commencement of TTM | A value of 1 is given if there is a TTM commencement objective and 0 if there is not |
| Servo-control use | A value of 1 is given if the temperature control is performed through catheters/hydrogel patches with advanced servo-control devices, and a 0 if the control is carried out by any of the other alternative methods provided in the survey. |
| Thermal recovery | A value of 1 is given if there is a recovery of the temperature after the TTM and 0 if there is not. |
| The aim is to reach a temperature | A value of 1 is given if there is a target temperature and 0 if there is not. |
| Use of 72-hour prognostic scales | A value of 1 is given if prognosis scales are applied during the first 72 hours and a 0 if they are not. |
| Long-term monitoring | A value of 1 is given if the patients are monitored in the long term and a 0 if they are not. |
| Rehabilitation program | A value of 1 is given if there is a rehabilitation protocol and 0 if there is not. |

PCI, percutaneous coronary intervention; TTM, targeted-temperature management

**Supplementary Table 3. Normalized proportion of patients in each neurological state at discharge according to the use of servo-control for temperature management**

|  | **CPC1** | **CPC2** | **CPC3** | **CPC4** |
| --- | --- | --- | --- | --- |
| **Without servo-control** | 0.379 | 0.248 | 0.217 | 0.156 |
| **With servo-control** | 0.471 | 0.251 | 0.143 | 0.135 |

**Supplementary Table 4. Average total cost per patient admitted with cardiac arrest according to the neurological outcome at discharge**

| **Neurological outcome** | **Average cost per patient (2020 Euros)** |
| --- | --- |
| CPC1 | 28,332.7 |
| CPC2 | 52,950.3 |
| CPC3 | 71,207.9 |
| CPC4 | 97,953.1 |

CPC, Cerebral Performance Category

**Supplementary Table 5. Average direct and indirect costs per hospital for patients admitted with cardiac arrest**

| **Average cost** | **2020 Euros** | **% of the**  **direct cost** | **% of the total cost** |
| --- | --- | --- | --- |
| Average ICU cost | 155,324 | 33.4 |  |
| Average cost for hospital stay | 131,975 | 28.4 |  |
| Average prognostication cost | 2,975 | 0.6 |  |
| Average rehabilitation cost | 141,026 | 30.3 |  |
| Average TTM cost | 33,581 | 7.2 |  |
| Average direct cost | 464,881 | 100 | 60.5 |
| Average indirect cost | 303,592 |  | 39.5 |
| Average total cost | 768,474 |  | 100 |

ICU, intensive care unit; TTM, targeted-temperature management

**
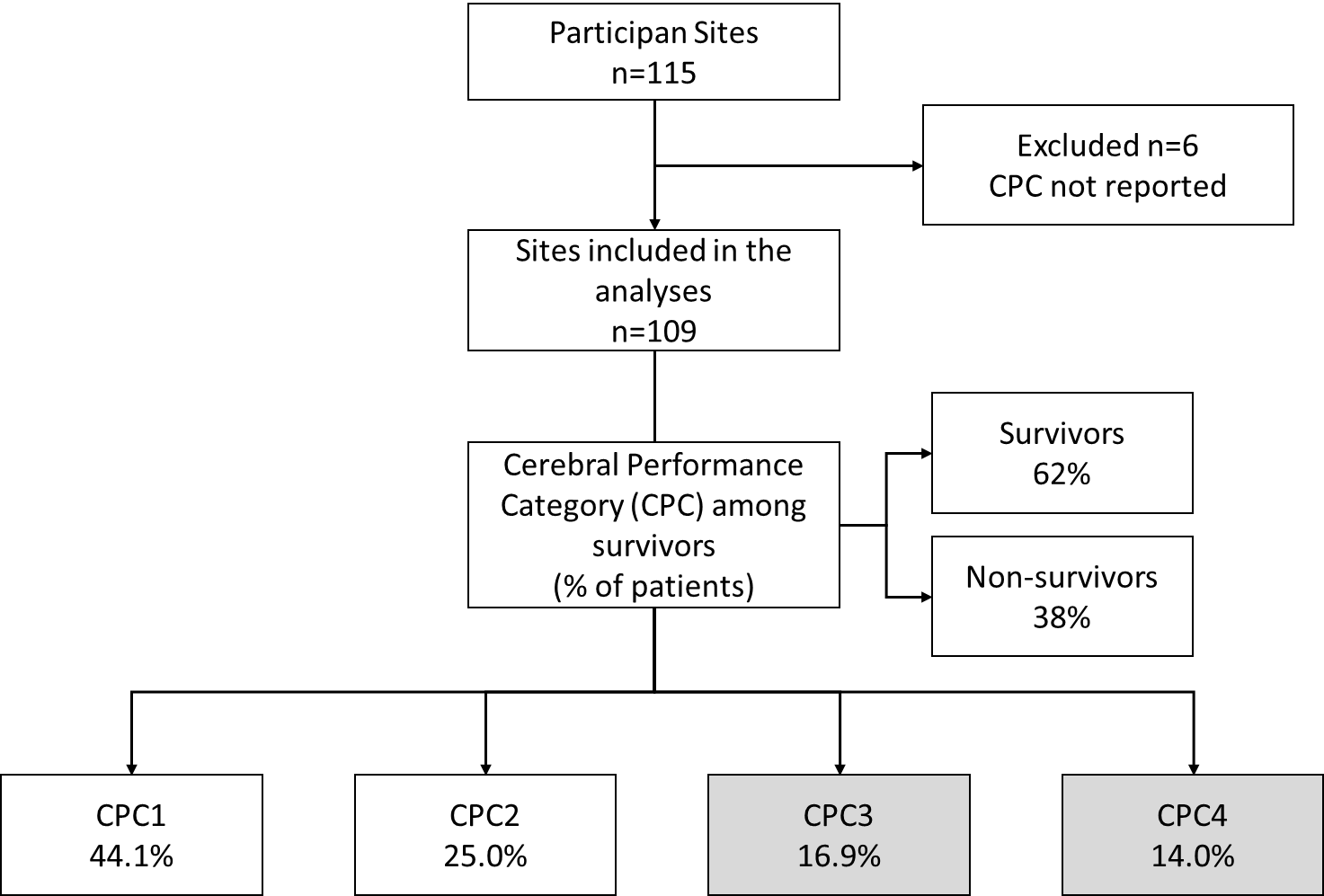
Supplementary Figure 1. Patient disposition**

| Supplementary Table 6. Results of variability analysis regarding the care of comatose patients after out-of-hospital cardiac arrest (OHCA) and return of spontaneous circulation (ROSC) | | |
| --- | --- | --- |
| **PROTOCOLS** | **Reply** | **% (n)** |
| Existence of post cardiac arrest protocol | Yes | 75.7 (87) |
| Protocols in your Unit | Organ donation | 91.3 (105) |
| Major importance protocol | Diagnostic approach protocol  Temperature control | 39.1 (45)  27.8 (32) |
| Hospital department receiving comatose patients recovering from out-of-hospital cardiac arrest (OHCA)  Presumed non-cardiac  Presumed cardiac | ICU/Critical Unit  ICU/Critical Unit | 67.0 (77)  52.2 (60) |
| Populations undergoing coronary angiography and emergent percutaneous coronary intervention (PCI) | Some  Any suspected of acute coronary syndrome | 76.5 (88)  71.6 (63) |
| Age limit for not performing coronary angiography and emergent PCI in comatose patients | Does not exist | 97.4 (112) |
| Time target for PCI | Yes  It is usually measured 61-120 minutes | 77.4 (89)  79.7 (71)  67.6 (48) |
| Average time PCI is performed | 120 minutes | 45.2 (52) |
| Conducting of regular audits | No | 51.3 (59) |
| Screening protocol for arrhythmogenic diseases | No | 43.5 (50) |

| Supplementary Table 7. Results of variability analysis on the structure of healthcare units (staff and tests available in the units). | | |
| --- | --- | --- |
| **UNIT STRUCTURES** | **REPLAY** | **% (n)** |
| PCI service in your centre Shift | Yes, 24 Hours, 7 days a week  Morning shifts from Monday to Friday | 66.1 (76)  40.0 (4) |
| Ultrasound service in your centre Shift | Yes, 24 Hours, 7 days a week  Morning shifts from Monday to Friday | 84.3 (97)  62.5 (10) |
| CT service in your centre Shift | Yes, 24 Hours, 7 days a week  Morning shifts from Monday to Friday | 99.1 (114)  100 (1) |
| EEG service in your centre Shift | Yes, but with restricted shift  Morning shifts | 70.4 (81)  36.4 (28) |
| Neurophysiology service in your centre Shift | Yes, but with restricted shift  Morning shifts from Monday to Friday | 75.7 (87)  29.7 (25) |
| Cardiology service in your centre Shift | Yes, 24 Hours, 7 days a week  Morning shifts | 65.2 (75)  39.4 (15) |

| Supplementary Table 8. Results of variability analysis on temperature control (temperature control management and protocols) | | |
| --- | --- | --- |
| **TEMPERATURE CONTROL** | **Reply** | **% (n)** |
| Active control of the temperature of comatose patients after out-of-hospital cardiac arrest with ROSC | Yes, for all patients | 67.8 (78) |
| Where does temperature control (TTM) start? | ICU | 70.4 (81) |
| Is TTM initiated before or after PCI | After PCI | 50.4 (58) |
| Time target for the start of TTM | No  It is usually measured  2h- 12h | 65.2 (75)  77.5 (31)  51.2 (20) |
| Means of temperature control | Hydrogel catheters/patches with advanced servo-control devices  Physical measures: cold compresses, fans/wet towels | 53.0 (61)  51.3 (59) |
| Temperature recovery after TTM | Yes  0.1 – 0.25oC/hour | 68.7 (79)  52.2 (60) |
| Written TTM protocol | Yes | 58.3 (67) |
| Target temperature | Yes  Fixed temperature  Fixed temperature 33ºC  Range 34-36ºC | 75.7 (87)  54.0 (47)  36.1 (17)  27.5 (11) |

| Supplementary Table 9. Results of variability analysis on prognosis (assessment of patients’ condition and follow-up) | | |
| --- | --- | --- |
| **PROGNOSIS** | **Reply** | **% (n)** |
| Application of prognostic scales in the first 72 hours | No | 67.8 (78) |
| Timing of the neuroprognosis | 72 hours after the arrest | 55,6 (64) |
| Protocol to limit therapeutic effort | Yes  Donation in end-of-life care | 80.0 (92)  100 (92) |
| Method of prognosis | Neurological examination  EEG | 96.5 (111)  92.2 (106) |
| Assessments performed in the neurological evaluation upon discharge | GOS (Glasgow Outcome Scale) | 61.7 (71) |
| Long-term follow-up of patients | No  Performed at the hospital  Every 6 months | 71.3 (82)  90.9 (30)  63.6 (21) |
| Rehabilitation protocol for patients | No locomotive rehabilitation  6 sessions on average, average duration of sessions 8 months | 58.3 (67)  95.4 (42) |
| Average percentage of patients per year according to neurological damage on discharge | CPC1 (without sequelae)  CPC2 (mild disability, is independent, does not require institutionalisation)  CPC3 (severe disability, not independent, requires institutionalisation)  CPC4 (persistent vegetative state)  CPC5 (death) | 26%  16%  13%  9%  36% |
| Average patient length of stay | In the ICU/CU  In the hospital | 10 days  25 days |
